# Age-correlated stress resistance improves fitness of yeast: support from agent-based simulations

**DOI:** 10.1186/1752-0509-8-18

**Published:** 2014-02-15

**Authors:** Ferdi L Hellweger, Neil D Fredrick, John A Berges

**Affiliations:** 1 Department of Civil and Environmental Engineering, Northeastern University, Boston, MA 02115, USA; 2 Department of Biological Sciences and School of Freshwater Science, University of Wisconsin-Milwaukee, Milwaukee, WI 53201, USA

**Keywords:** Agent-based modeling, Bet hedging, Stress resistance, Yeast

## Abstract

**Background:**

Resistance to stress is often heterogeneous among individuals within a population, which helps protect against intermittent stress (bet hedging). This is also the case for heat shock resistance in the budding yeast *Saccharomyces cerevisiae*. Interestingly, the resistance appears to be continuously distributed (vs. binary, switch-like) and correlated with replicative age (vs. random). Older, slower-growing cells are more resistant than younger, faster-growing ones. Is there a fitness benefit to age-correlated stress resistance?

**Results:**

Here this hypothesis is explored using a simple agent-based model, which simulates a population of individual cells that grow and replicate. Cells age by accumulating damage, which lowers their growth rate. They synthesize trehalose at a metabolic cost, which helps protect against heat shock. Proteins Tsl1 and Tps3 (trehalose synthase complex regulatory subunit TSL1 and TPS3) represent the trehalose synthesis complex and they are expressed using constant, age-dependent and stochastic terms. The model was constrained by calibration and comparison to data from the literature, including individual-based observations obtained using high-throughput microscopy and flow cytometry. A heterogeneity network was developed, which highlights the predominant sources and pathways of resistance heterogeneity. To determine the best trehalose synthesis strategy, model strains with different Tsl1/Tps3 expression parameters were placed in competition in an environment with intermittent heat shocks.

**Conclusions:**

For high severities and low frequencies of heat shock, the winning strain used an age-dependent bet hedging strategy, which shows that there can be a benefit to age-correlated stress resistance. The study also illustrates the utility of combining individual-based observations and modeling to understand mechanisms underlying population heterogeneity, and the effect on fitness.

## Background

There is increasing appreciation for individuality of microbes [1,2]. Even populations grown up from a single cell, in a constant environment can exhibit significant phenotypic heterogeneity in gene expression, protein content and physiology. Individual heterogeneity can be important to population fitness by allowing different functions (e.g. C and N fixation in filamentous cyanobacteria) and survival in a fluctuating environment. One prominent example is bacterial persistence, where a typical population contains a small fraction of slow- or non-growing “persister” cells that are not killed by antibiotics [3,4]. Cells switch between normal and persister states in a random, binary (switch-like) manner. The ability to resist stress comes at a cost (typically reduced rates of growth or reproduction). For intermittent stress, there is an advantage to maintaining heterogeneity among individuals in a population in terms of tradeoffs between performance and survival (i.e. an insurance mechanism referred to as bet hedging [3,5]).

For eukaryotic microbes, the budding yeast *Saccharomyces cerevisiae* (*S. cervisiae*) is a model organism for studying individual heterogeneity and aging and longevity [1,6]. Various mechanisms, including stochastic variability in regulatory pathways and production/destruction of mRNAs, and deterministic asynchronicity in cell cycle or replicative age, lead to heterogeneity in protein content and stress resistance in clonal populations [1,7-12]. For example, copper resistance is heterogeneous and related to cell cycle and replicative age [13]. Intrinsic and induced expression of heat shock proteins and resistance is heterogeneous [11,14]. Expression of Tsl1, used in the synthesis of trehalose (an alpha-linked disaccharide of glucose that enhances thermotolerance, reduces aggregation of denatured proteins and protects against oxidative damage), and heat shock resistance are heterogeneous and correlated with replicative age [15]. Natural yeast (i.e. not *S. cervisiae*) populations were found to have heterogeneous resistance to copper, lead and sulfur dioxide, and this phenotypic heterogeneity is a beneficial and evolvable trait [16]. Unlike in bacteria, heat shock resistance in yeast is graded, continuous (vs. binary) and correlated with replicative age (vs. random).

The benefit of random heterogeneous expression of a stress-response factor has been demonstrated experimentally and computationally for *S. cervisiae*[8]. Levy et al. [15] hypothesized that correlating heat shock resistance with age provides an added benefit. The idea is that older, slower-growing cells are better candidates for being stress resistant because they contribute relatively less to the population growth. Is there a fitness benefit to age-correlated (vs. random) stress resistance?

This hypothesis is explored here using modeling, an approach that has been applied previously to explore the role of heterogeneity [5,17]. The general strategy is to develop a mathematical model that includes the relevant mechanisms, and then perform numerical competition experiments to see if the age-correlated resistance trait is beneficial. Such competition and/or evolutionary optimization simulations have been used previously to determine optimal traits/parameters [18-20]. There are many potential model formulations and associated parameter sets for simulating replication, aging, resistance, etc. (reviewed below). To ensure some degree of realism, we constrain the model by calibration and comparison to relevant observations from the literature. We use agent-based modeling (ABM, aka individual-based modeling, IBM), rather than the more common population-level modeling approach [18-22]. An ABM is appropriate in this case, because it can resolve the continuous/graded distribution of various individual properties (e.g., protein levels, growth rate, resistance), and model outputs can be compared directly to the individual-based observations that are used to constrain the model [15,23]. The model simulates intracellular mechanisms and the cell behavior emerges (systems biology). Then, it simulates many such cells and the population behavior emerges (systems ecology). This multi-level approach has been referred to as 'systems bioecology’ [19-21].

We describe the model and compare it to data from the literature, which shows that it is generally consistent with the observed patterns. A heterogeneity network is developed, which highlights the predominant sources and pathways of resistance heterogeneity. Then we perform competition experiments with strains that have different Tsl1/Tps3 expression strategies in an environment with intermittent heat shocks. For conditions with high severity and low frequency of heat shocks, an age-dependent bet hedging strategy is most beneficial, which supports the hypothesis of a fitness benefit of age-correlated stress resistance.

## Methods

### Model overview

**Figure 1:**
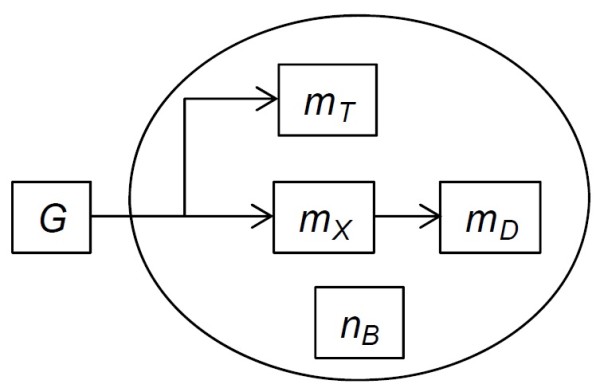
**Model schematic.** Symbols: *G* = glucose, *m*_*X*_ = structural mass, *m*_*D*_ = damaged mass, *m*_*T*_ = trehalose mass and *n*_*B*_ = number of bud scars.

The model is relatively simple and resolves only those mechanisms necessary for exploring the hypothesis and comparison to the relevant data. Yeast cells take up glucose (*G*, g L^-1^) and convert it to biomass (Figure 1). Three forms of biomass are considered, including structural (*m*_*X*_, g dry cell^-1^), damaged (*m*_*D*_, g dry cell^-1^) and trehalose (*m*_*T*_, g dry cell^-1^). The total biomass (*m*, g dry cell^-1^) is the sum of these components (*m* = *m*_*X*_ + *m*_*D*_ + *m*_*T*_). Structural biomass becomes damaged. A fraction of biomass is synthesized as trehalose. The model tracks the age or number of divisions in terms of bud scars (*n*_*B*_). A population of individual cells is simulated using an agent-based approach.

### Biomass growth

A number of metabolism models for *S. cervisiae* have been developed ranging from simple Monod-type growth equations to more detailed kinetic models that resolve intracellular mechanisms up to dynamic/kinetic implementations genome-scale network reconstructions [24-28].

Here, a relatively simple approach is used, with the mass balances for the three components:

(1a) $$\frac{dm_{X}}{\mathrm{dt}}=\left(1-f_{s}\right)\;\mu \;m_{X}-k_{d}\;m_{X}$$

(1b) $$\frac{dm_{D}}{\mathrm{dt}}=k_{d}\;m_{X}$$

(1c) $$\frac{dm_{T}}{\mathrm{dt}}=f_{s}\;\mu \;m_{X}$$

where *μ* (day^-1^) is the growth rate, *f*_*s*_ is the trehalose synthesis fraction and *k*_*d*_ (day^-1^) is the damage rate. The growth rate increases with glucose and decreases with damage:

(2) $$\mu =\mu_{m,g}\frac{G}{K_{g}+G}\;\frac{K_{d}^{n_{d}}}{K_{d}^{n_{d}}+{\left(m_{D}/m\right)}^{n_{d}}}$$

where *μ*_*m,g*_ (day^-1^) is the maximum growth rate, *K*_*g*_ (g L^-1^) is the half-saturation constant for glucose, *K*_*d*_ is the half-saturation constant and *n*_*d*_ is the exponent for damage. The model dynamically simulates the extracellular glucose concentration considering inflow and washout (for continuous culture simulations) and uptake by the cells (see Additional file 1: SI text for details).

### Replication

Cell division in *S. cervisiae* is via the asymmetrical budding process, where a larger mother cell gives birth to a smaller daughter cell. With subsequent births, the mother’s size increases and it accumulates bud scars and damage (see below). A number of cell cycle and replication models for *S. cervisiae* have been developed [29,30].

Here, the model of Vanoni et al. [30] is adopted. Briefly, two cell cycle phases are simulated, unbudded and budding. Budding starts at a threshold budding size (*m*_*b*_), which increases from a specified daughter cell value (*m*_*b,0*_) by a factor ($a_{m}\;{b_{m}}^{n_{B}-1}$) with each generation. Division occurs at a threshold replication size (*m*_*r*_), which is proportional to the budding size (*m*_*r*_ = *f*_*m,r*_*m*_*b*_). The daughter gets the mass synthesized during the budding phase. The mother gains a bud scar and preferentially retains the damage (see below). Individual phenotypic heterogeneity is introduced by randomizing the budding size. At birth, the daughter’s budding size is drawn from a global truncated normal distribution with specified mean and coefficient of variation (CV) [31]. A truncated distribution is used to prevent unrealistic values (e.g., *m*_*b,0*_ < 0). This process introduces non-heritable phenotypic heterogeneity, so values are drawn from a global distribution (vs. one with the mean based on the mother).

### Aging and damage accumulation

Aging in *S. cervisiae* is due to a number of mechanisms, including accumulation of extrachromosomal DNA circles (ERCs) and oxidative damage (e.g., carbonylation) to proteins [32]. At division, this damage is preferentially retained by the mother cell, although the ability to do so diminishes with replicative age [33,34]. In addition, the mother cell has higher reactive oxygen species (ROS) and protein damage rates and lower damaged protein degradation rates [35,36]. Trehalose protects against ROS damage to proteins [37]. Several generic models of aging have been presented [18,34]. Specifically for *S. cervisiae*, Hirsch [38] developed a model where cells accumulate a senescence factor at a constant rate and partitions it asymmetrically at division. The growth rate decreases with increasing amount of this senescence factor. More mechanistic models that explicitly represent ROS, the damage reaction with a protein (citrate synthase) and repair reaction with a heat shock protein (Hsp90) have been presented [39].

The present model considers the production of damaged mass (*m*_*D*_) from structural mass (*m*_*X*_) in a first-order manner, at a damage rate that increases with age ($k_{d}=a_{d}\;{n_{B}}^{b_{d}}$). At division, the damage mass is preferentially retained by the mother, based on a split fraction (*s*_*d*_).

### Trehalose synthesis and Tsl1/Tps3 expression

Trehalose serves as storage carbohydrate and stress protectant [40-42]. Synthesis is highest during the stationary phase and in response to stress (incl. heat), but trehalose also accumulates under normal, non-stressed conditions [42-44]. Trehalose is synthesized by a trimeric protein complex made up of Tps1 and Tps2, and interchangeable Tps3 or Tsl1 [45,46]. Genes involved in trehalose synthesis are induced by heat shock [46,47]. In addition, the expression is negatively correlated with growth rate and has a stochastic component [15,47]. Tsl1 and Tps3 promoters share a common regulatory element (stress-responsive element, STRE), but their expression can differ [9,10,46]. Trehalose (or more generally carbohydrate storage) has been included in metabolic models of *S. cervisiae*[24,25,48].

In the present model, the trehalose synthesis complex is represented by Tsl1 and Tps3, which are assumed to limit the trehalose synthesis rate (i.e., Tps1 and Tps2 are assumed to be present in excess). Tsl1 and Tps3 are considered separately (vs. the complete synthase complex or Tps1), because their expression differs and to allow for direct comparison to observations [15,46]. The trehalose synthesis fraction (*f*_*s*_) is a function of the total trehalose synthesis enzyme concentration (*e*_*s*_ = *e*_*Tsl1*_ + *e*_*Tps3*_):

(3) $$f_{s}=f_{m,s}\frac{e_{s}^{n_{s}}}{K_{s}^{n_{s}}+e_{s}^{n_{s}}}$$

where *f*_*m,s*_ is the maximum fraction, *K*_*s*_ (mmol L^-1^) is the half-saturation constant and *n*_*s*_ is the exponent for trehalose synthesis.

Tsl1 and Tps3 are expressed using a set of constant, age-dependent and stochastic terms. In the model applications, cells are grown in constant, glucose-replete conditions, so the effect of growth condition (i.e., stationary phase) on expression is not included. Cells are subjected to heat shock, but since the observed resistance was not due to an induced heat shock response [15], induction of resistance by heat shock is not included. The Tsl1 enzyme concentration (*e*_*Tsl1*_, mol L^-1^) is assumed to adjust rapidly to the damage according to:

(4) $$e_{\mathrm{Tsl}1}=\left[e_{c,\mathrm{Tsl}1}+e_{a,\mathrm{Tsl}1}\;\frac{m_{D}/m}{K_{\mathrm{Tsl}1}+m_{D}/m}\right]\;f_{r,\mathrm{Tsl}1}$$

where *e*_*c,Tsl1*_ (mol L^-1^) is the magnitude of constant expression, *e*_*a,Tsl1*_ (mol L^-1^) is the magnitude of damage or age-dependent expression, *K*_*Tsl1*_ is a half-saturation constant and *f*_*r,Tsl1*_ is a randomization factor. Note that expression is a function of the combined effect of constant, age-dependent and stochastic terms, with their relative contribution depending on the assigned parameter values. The randomization factor (*f*_*r,Tsl1*_) is varied by drawing from a global truncated normal distribution with mean of 1.0 and specified CV, following the same approach used for *m*_*b,0*_ (see above). An equivalent formulation is used for Tps3.

### Heat shock tolerance and death

Heat causes denaturation of proteins and there are a number of mechanisms that can prevent this. Trehalose stabilizes proteins during heat shock [40]. Other factors include various heat shock proteins, whose intrinsic (i.e. without heat shock) expression is also heterogeneous and correlated with heat shock resistance [11,14].

Mortality by heat shock is simulated using a deterministic approach [8]. Specifically, the applied heat shock severity (*H*_*a*_, arbitrary units) is compared to the tolerance of the cell (*H*_*t*_), which increases with the trehalose mass fraction (*m*_*T*_ / *m*):

(5) $$H_{t}=\frac{m_{T}/m}{K_{h}+m_{T}/m}$$

where *K*_*h*_ is the half-saturation constant for heat shock tolerance. *K*_*h*_ is the fraction of trehalose required to achieve a tolerance of 0.5. When a heat shock is applied, all cells with *H*_*t*_ < *H*_*a*_ die. *H*_*a*_ can be adjusted to reflect different experimental conditions.

### Additional mechanisms of heterogeneity

The model includes a number of deterministic sources of heterogeneity, like the uneven split of damage among mother and daughter at replication. Also, the budding mass threshold (*m*_*b,0*_) and Tsl1 and Tps3 expression factors (*f*_*r,Tsl1*_ and *f*_*r,Tps3*_) are varied stochastically, as described above. However, in reality there are numerous other mechanisms (e.g., stochastic expression of all genes) that contribute to heterogeneity in cellular processes [1,7-12]. To account for this, the maximum growth rate (*μ*_*m,g*_) and damage exponent (*n*_*d*_) parameters are also randomized (following the same approach used for *m*_*b,0*_).

### Agent-based modeling of population

The model simulates individual yeast cells using an agent-based approach [18-22]. Each agent stores the cell state variables (e.g., *m*_*X*_, see Figure 1) and those parameters that are varied at the individual level (e.g., *m*_*b,0*_). For continuous culture simulations, the model includes stochastic washout of cells from the reactor (see Additional file 1: SI text for details). Differential equations (e.g., Eq. 1) are solved using an explicit numerical integration method. The model is implemented in the IAM framework [19,20], and the source code is available from the corresponding author.

For some experiments the model explicitly simulates each individual cell. This includes the microcolony experiments that have ~10^5^ colonies of up to ~100 cells for a total of 10^7^ cells (e.g. Additional file 1: Figure S1B). However, for liquid cultures with larger populations, including the competition experiments, this is not feasible. For example, a 300-mL culture with a cell density of 2.6 × 10^8^ cells mL^-1^ contains 7.9 × 10^10^ cells. Using the present model, simulating that many cells for 20 days would take approximately 15 years of CPU time and 18 PB of RAM memory. As is common in microbe ABMs, for liquid culture, the model simulates super-individuals, which are representative of a number of real individuals [21]. Minimum/maximum numbers of agents are specified, and when the number of agents drop/rises below/above this, agents are split/combined (see Additional file 1: SI text for details). The number of agents, or the computational resolution, is set sufficiently high so that the model produces robust and reproducible results over multiple runs with different seed values for the random number generator.

This application is especially challenging from a computational perspective, because of the focus on small fractions of the population. For example, in one experiment by Levy et al. [15], 0.1% of the population was sorted out using flow cytometry and the growth rate distribution of that fraction was computed (Additional file 1: Figure S1I). In order for the model to adequately resolve the heterogeneity of such a small fraction of the population, it needs to have a very large number of agents.

### Simulations performed

The model was constrained by calibration and comparison to relevant observations from the literature. Several parameters were calibrated within the available literature range with the help of an automated optimization routine (see Additional file 1: SI text, Table S1, Figures S1&2). Model simulations followed the actual experimental protocols as described in the respective literature references, which could be quite involved. For example, one experiment by Levy et al. [15] included growing cells in liquid suspension, sub-sampling based on Tsl1 expression, growing again in liquid suspension, randomly sub-sampling, growing as microcolonies, and estimating the growth rate of each microcolony based on the change in colony area (Additional file 1: Figure S1J). The resulting calibrated model is then used without any further changes to explore the underlying mechanisms and fitness effect of heterogeneous, age-correlated heat shock resistance.

**Figure 2:**
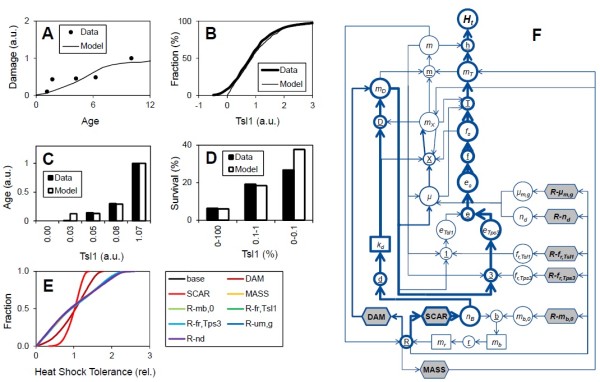
**Model-data comparison and heterogeneity source and pathway analysis. (A)** Damage (*m*_*D*_/*m*) vs. age (*n*_*B*_). Data from [33]. **(B)** Tsl1 expression (*e*_*Tsl1*_) distribution of cells. **(C)** Age (*n*_*B*_) vs. Tsl1 expression (*e*_*Tsl1*_). **(D)** Heat shock survival of various Tsl1-sorted fractions. Data from [15]. “a.u.” is arbitrary units. **(E)** Distribution of heat shock tolerance for base case and various diagnostic simulations (e.g. “dam” has equal damage partitioning, *s*_*d*_ = 0.5). **(F)** Heterogeneity network. Line weight indicates contribution of node or link to overall heterogeneity in heat shock tolerance (based on variance of normalized *H*_*t*_, e.g., panel **E**). For details of experiments used to generate the data the reader is referred to the source publications.

To understand how heterogeneity is produced and how it propagates through the population, we developed a heterogeneity network (Figure 2F). The nodes in the network represent individual state variables (e.g. *m*_*D*_), calculated variables (e.g. *μ*) and processes (e.g. unequal split of damage, node DAM), and the links represent causal relationships. For example, DAM causes heterogeneity in *m*_*D*_, which in turn causes heterogeneity in *m* (via mass summation, node m), *μ* (via Eq. 2), and *e*_*Tsl1*_ and *e*_*Tps3*_ (via Eq. 4). By turning off the heterogeneity at a node or link in the network and examining the resulting reduction in heterogeneity at a downstream node, the heterogeneity can be mapped onto the network (see Results section).

To explore the role of the age-correlated resistance trait on the fitness of the yeast, numerical (i.e. simulations) competition experiments were performed. Cells were grown in a glucose-limited chemostat with constant dilution rate (*D* = 0.15 h^-1^), a set-up similar to the one used in a previous experimental study that examined the effect of mild heat shock (28 > 36°C) on *S. cervisiae* growth rate and gene expression [47]. The culture was subjected to heat shocks at specified heat shock severity (*H*_*a*_) and frequency (*F*_*h*_, h^-1^). A number of model strains with different Tsl1/Tps3 expression parameters were developed. For Tsl1, *e*_*c,Tsl1*_ controls constant expression, *e*_*a,Tsl1*_ controls age-dependent expression, and *f*_*r,Tsl1,CV*_ controls stochasticity. Tsl1 and Tps3 are considered separately in the model to allow for comparison to data (Figure 2), but their effect on trehalose synthesis is identical (Eq. 3) and a strain with a high Tsl1 parameter (e.g., *e*_*c,Tsl1*_) behaves the same as a strain with an equivalently high Tps3 parameter (i.e., *e*_*c,Tps3*_). Therefore, the Tsl1 and Tps3 parameters were varied together (e.g., the ratio *e*_*c,Tps3*_ / *e*_*c,Tsl1*_ is held constant). We created 1,000 model strains, with 10 variants for each Tsl1 parameter. Doing a single simulation with all strains was not feasible. Therefore, a tournament-style competition was used. In each round, 10 strains were randomly selected and placed in competition. The winner of each match advanced to the next round and the process was repeated until one strain was left. For each condition (*H*_*a*_, *F*_*h*_), the optimal bet hedging strategy/parameters emerged as the winner. The experiment is implicitly constrained by the metabolic cost of trehalose synthesis (reduced synthesis of structural biomass, Eq. 1a), which is traded off against the benefit of higher heat shock survival.

## Results and discussion

### Calibration and comparison to data

The model was calibrated to observations from the literature with the help of an automatic calibration routine. The database is comprised of 15 datasets [9,10,15,33,41,43-45,49]. The reader is referred to the original publications for experimental protocols and details. This database characterizes the relevant features of the system, including the distribution of growth rates, damage accumulation with age, Tsl1 and Tps3 expression levels, distribution of Tsl1 expression, age vs. Tsl1 expression, age distribution for all and top 1% of Tsl1 expressing cells, Tsl1 expression vs. growth rate, growth rate distributions for all, top 1% and 0.1% of Tsl1 expressing cells, trehalose content, trehalose in wild type vs. Tsl1 knockout, survival vs. Tsl1 expression, and survival vs. growth rate for wild type and Tsl1 knockout strains. Discussion of all datasets is provided in the SI and a selected subset are discussed here.

Oxidative protein damage (carbonyl levels) was observed to increase with age (bud scars), and the model reproduces this general pattern (Figure 2A). The observations suggest a step-wise increase whereas the model exhibits a more linear shape. The reason for the discrepancy is unclear. The observations are from a single study and it would be useful to obtain additional observations to confirm the shape. Damage mass increases with age due to preferential inheritance by the mother and an increase of damage rate with age. The expression of Tsl1, observed with flow cytometry and green fluorescent protein (GFP) fused to Tsl1, was quite heterogeneous (Figure 2B). The modeled Tsl1 distribution was not as spread out as the observations, for example, the data extended into the negative range (presumably a measurement error at low Tsl1 levels), whereas the model restricted Tsl1 to the positive values. The heterogeneity in Tsl1 is a function of the stochastic component and the amount of Tsl1 expression that is damage correlated (Eq. 4). Applying the bud scar stain WGA-TRITC and passing cells through the flow cytometer showed that Tsl1 expression increased with age (Figure 2C). The model also shows an increase. However, for an unknown reason it over-predicts the age in the 0.03 Tsl1 bin. When Tsl1-sorted sub-populations were heat shocked, survival correlated positively with Tsl1 expression (Figure 2D). Again, the model reproduces the general increase, but it differs in the 0–0.1% Tsl1 bin. This narrow bin includes the fewest cells and is most susceptible to stochastic variability (observations and model). Overall, the model does not capture all features of the data, but it reproduced the main patterns, including an increase of damage with age, heterogeneous Tsl1 expression, and correlation of age and survival with Tsl1 expression.

### Source and pathway of heterogeneity

We constructed a heterogeneity network (Figure 2F), which defines how heterogeneity can be produced and propagate through the population. In the present model, all heterogeneity originates at replication. The model does not consider other sources of heterogeneity, like stochastic differentiation at other times (i.e. in between replication events) or heterogeneity in the environment. There are a number of deterministic and stochastic sources of heterogeneity associated with replication (grey nodes in Figure 2F). For example, the scarring process produces heterogeneity in bud scars (*n*_*B*_) in a deterministic manner, while the expression of Tsl1 is varied randomly (*f*_*r,Tsl1*_). Despite the numerous sources of heterogeneity, replication does not completely randomize or “reset” the cells, and the model allows for inter-generation memory. For example, bud scars and damage – and thus also the growth rate – are heritable (Additional file 1: Figures S1A&S3).

Where does the heterogeneity in survival originate? Sequentially removing sources and examining the resulting reduction of heterogeneity in heat shock tolerance showed that the scarring and unequal division of damage processes are the predominant sources (Figure 2E). But there are many ways the heterogeneity can go from these sources to heat shock tolerance. How does it propagate through the network? Systematically eliminating heterogeneity at links and nodes in the network (e.g., use population-average *e*_*S*_ in Eq. 3) allowed us to map the heterogeneity onto the network. This showed that heterogeneity travels along multiple pathways, but predominantly from scarring to damage to Tps3 expression to trehalose and heat shock tolerance, a deterministic pathway that leads to age-correlated stress resistance.

### Competition experiments

**Figure 3:**
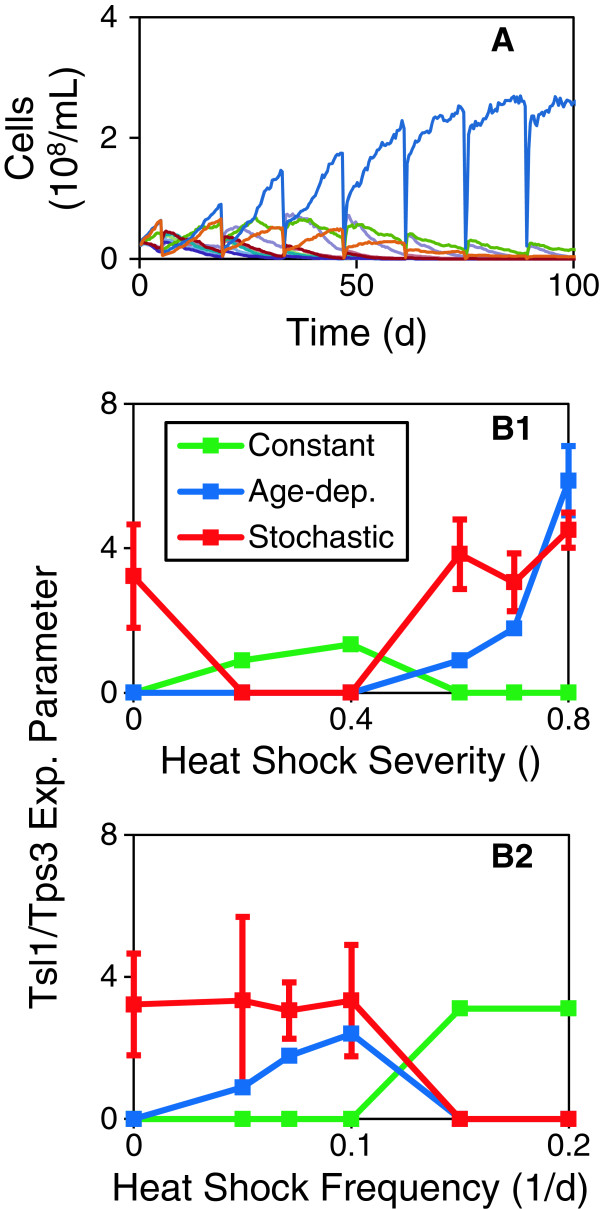
**Numerical competition experiments. (A)** Example of one simulation at intermediate heat shock severity and frequency (*H*_*a*_ = 0.7, *F*_*h*_ = 0.14 d^-1^). Cell density of 10 competitors. Abrupt drops in concentrations correspond to heat shocks. **(B)** Summary of tournament-style competitions. Optimal Tsl1/Tps3 expression parameters for a number of (B1) heat shock severities (*H*_*a*_ varies, *F*_*h*_ = 0.14 d^-1^) and (B2) frequencies (*H*_*a*_ = 0.7, *F*_*h*_ varies). Constant: *e*_*c,Tsl1*_ in μM, Age-dependent: *e*_*a,Tsl1*_ in μM, Stochastic: *f*_*r,Tsl1*_ / 10. Symbols are mean +/- one standard deviation of ten replicate experiments.

The model did not capture all features of the data, but it reproduced the major patterns observed in the relevant datasets. It was then used as an experimental system to explore the hypothesis outlined in the introduction. To determine the best Tsl1/Tps3 expression strategy we performed tournament-style competition experiments between 1,000 strains with different expression parameters (*e*_*c,Tsl1*_, *e*_*a,Tsl1*_, *f*_*r,Tsl1,CV*_) in continuous culture with intermittent heat shocks. Figure 3A shows the results from one simulation at intermediate heat shock severity and frequency. One strain clearly outcompeted the others over the course of the experiment.

We performed a number of experiments at various heat shock severities (*H*_*a*_) and frequencies (*F*_*h*_), ranging from no heat stress (*H*_*a*_ = *F*_*h*_ = 0) to the maximum heat stress the yeast can survive (Figure 3B). When no heat shocks were applied, the winning strain had no constant or age-dependent terms. It did have a stochastic term, but this is simply due to the neutrality of the parameter when constant and age-dependent terms are zero (i.e., then the stochastic parameter does not affect the expression, Eq. 4). At lower heat shock severities, the winning strategy was to express Tsl1/Tps3 in a constant manner without heterogeneity (Figure 3B1). It only takes a small amount of trehalose to survive these heat shocks and it is best to have all cells synthesize this amount. Adding heterogenity would result in some cells being killed by heat shock, which would not be beneficial, a finding consistent with previous studies [8]. Since heterogeneity cannot be avoided with age-dependent expression, constant expression is the better strategy in this case. At higher severities, the amount of trehalose required to survive the heat shocks becomes larger and a bet hedging strategy becomes beneficial. That is, the average amount of trehalose is below what is required to survive the heat shocks, but it is heterogeneous and some cells have sufficient trehalose to survive the heat shocks, and this prevents the population from being wiped out. Under such conditions, the model predicted that age-dependent expression is better. This can be explained, as suggested in the Introduction, by the fact that the older cells contribute less to the population growth, and eliminating them is less detrimental to population growth (Additional file 1: Figure S11). If age-dependent expression is excluded (*e*_*a,Tsl1*_ = 0), the winning strain has constant and stochastic expression terms (Additional file 1: Figure S10). The heterogeneity introduced through the deterministic aging process is sub-optimal and it is beneficial to add more via the stochastic term. At lower heat shock frequencies, the winning strategy was age-dependent bet hedging, whereas at higher frequencies constant expression without heterogeneity was better (Figure 3B2). At lower frequencies, the growth period in between the heat shocks is relatively long and reducing the average trehalose production (as is achieved using a bet hedging strategy) is beneficial. At higher frequencies, a bet hedging strategy is not advantageous, because too many cells are lost through the frequent heat shocks.

These experiments were performed with a model designed with equations based on our current understanding of the underlying mechanisms, a parameter set that is generally consistent with the literature and main patterns consistent with observations. However, we cannot rule out that there is not another model formulation (i.e. different equations) or parameter set that produces an equal-quality calibration but a different result or conclusion about the fitness effect of age-correlated heat shock resistance. This is a common problem in model prediction and has been referred to as “equifinality” [50], and it can be addressed to some extent by varying model formulations [18,34] and/or parameters [29]. The present model is computationally very demanding. Nonetheless, we used an automated optimization routine that allows for alternate parameter values, and two runs produced essentially the same parameter set, which provides some additional support for the robustness of our conclusions.

## Conclusion and outlook

This study explored the ecological role of heterogeneous, age-correlated heat shock resistance in *S. cervisiae*. A simple model was constructed based on our current understanding of the underlying mechanisms, and comparison to relevant data shows it is consistent with observed patterns. Competition experiments with strains that have different stress protectant synthesis strategies shows that, for high severities and low frequencies of heat shock, an age-dependent bet hedging strategy is best. This supports the hypothesis that age-correlated resistance is more beneficial than random resistance. Although the model is specific to heat shock resistance in *S. cervisiae*, trehalose is produced by many different organisms and also protects against other forms of stress (e.g., ethanol, [41]), so our results have broader relevance. However, there are also cases where resistance is negatively correlated with age (i.e., younger cells are more resistant), like Sod1p-mediated copper resistance [13], so these results cannot be generalized to all types of stress.

The finding that it is advantageous for older cells to invest in increasing stress tolerance has implication for understanding aging and longevity- two very different things, with different selective forces acting [6]. Longevity is a highly adaptive trait and it is generally considered that genes promoting longer lifespan do so by improving somatic resistance in unfavorable conditions [6]. Our results provide a clear example of how such a mechanism could operate.

Our model was designed specifically for exploring the role of age-correlated heat shock resistance in *S. cervisiae*. For that purpose it was kept as simple as possible, while still including the relevant mechanisms. This naturally limits the model’s applicability to other questions, although it should be useful for exploring other features related to aging, heterogeneity and stress resistance. For example, with minimal changes (i.e. Eq. 4), the present model could be used to predict expression of other proteins. The model can also serve as a stepping stone for further model development. A lot more is known about the various mechanisms involved in the problem and this knowledge is sufficient to support the development of a more detailed model. It would be interesting to bring in more mechanistically-detailed models of gene transcription and expression noise [7,8,12], more detailed and/or genome-scale metabolism [24,26-28] and cell cycle control [29]. Sub-genome scale combined signaling, gene expression and metabolism models have been developed [51]. It seems that several pieces are in place to support the development of such a model, which would require a large community effort (as was done for the latest metabolic network reconstruction, [27]), but it would be worth it.

This study combined individual-based observations (IBO) and modeling (IBM) to understand mechanisms underlying population heterogeneity, and the effect on fitness [23]. Individual-based observation and experimental technologies are advancing rapidly and are generating large amount of novel data [15,52]. These data are different from traditional population-level observations, which were amenable to analysis using traditional population-level models, and they require new methods and models. Our study illustrates the utility of combining IBM and IBO. The IBOs of Levy et al. [15] were used to constrain the individual-level processes in the IBM. The IBM, in turn, put the IBOs into ecological context.

This paper presents the use of a multi-scale modeling approach to investigate the role of an intracellular mechanism in the ecological fitness of an organism. Covering multiple levels of organization is a general problem in the biological sciences. Several systems approaches have been developed to address this challenge [53,54]. The approach used here, “systems bioecology”, combines systems biology and systems ecology [19-21]. The idea is conceptually quite simple. First, the intracellular states and mechanisms of microorganisms are explicitly simulated (systems biology). Then, whole populations of these individual microbes are simulated directly using agent-based modeling (ABM), including their interaction with the environment (systems ecology). This general approach may be applicable to other questions involving the role of intracellular mechanisms at the ecosystem scale.

## Competing interests

The authors declare that they have no competing of interests.

## Authors’ contributions

FLH and JAB designed the experiment. FLH and NDF performed the research. FLH and JAB wrote the paper. All authors read and approved the final manuscript.

## Supplementary Material

Additional file 1 **Additional model details.** Additional discussion of model results. Click here for file
