# Supplementary material for: Age-correlated stress resistance improves fitness of yeast: support from agent-based simulations
Source: BMC Syst Biol. 2014 Feb 15;8:18. doi: 10.1186/1752-0509-8-18 (PMC3927587; doi:10.1186/1752-0509-8-18)
Supplement: Additional file 1 — Additional model details. Additional discussion of model results. [file 1752-0509-8-18-S1.docx]

**Age-correlated stress resistance improves fitness of yeast: support from agent-based simulations**

Ferdi L. Hellweger^1,*^, Neil D. Fredrick^1^, John A. Berges^2^

^1^Dept. of Civil and Environmental Engineering and Center for Urban Environmental Studies, Northeastern Univ., Boston, MA 02115

^2^University of Wisconsin-Milwaukee, Department of Biological Sciences and School of Freshwater Science, Milwaukee, WI 53201

^*^Corresponding author: ferdi@coe.neu.edu

**Supplementary Information**

S1. Additional model details

*Environment simulation*

In continuous culture, cells can be washed out of the reactor at a probability of *Q* / *V_R_* *Δt*, where *V_R_* (L) is the reactor volume, *Q* (L h^-1^) is the flow rate and *Δt* (h) is the time step.

Glucose is supplied by the inflow, washed out and taken up by yeast. The glucose mass balance is:

$$V_{R} \frac{dG}{dt}=Q \left(G_{IN}-G\right)-\sum_{cells} \frac{\mu}{Y_{g}} m_{X}$$

(S1)

where *G_IN_* and *G* (g L^-1^) are the inflow and reactor glucose concentrations, respectively, and *Y_g_* is the yield coefficient.

*Agent-accounting in competition simulation*

For liquid culture experiments with large number of cells, the model simulates agents or "super-individuals", as done in previous microbe ABMs [[1-3](#_ENREF_1)]. Each agent is representative of *S_R_* cells. The number of agents (*n*) is kept within a user-specified range (*n_MIN_* - *n_MAX_*) by a split/combine routine. If *n* decreases below the *n_MIN_*, the agent with the highest *S_R_* is split into two identical agents with half *S_R_* each. If *n* increases above *n_MAX_*, the two agents with the lowest *S_R_* are combined. Of these two agents, the one with the lower *S_R_* is discarded and the *S_R_* of the agent with the higher *S_R_* is increases to conserve the number of cells. The model tracks multiple strains and maintains the number of agents within each strain between *n_MIN_* and *n_MAX_* [[1](#_ENREF_1), [4](#_ENREF_4)].

*Automated optimization routine*

Several parameters were calibrated within the available literature range with the help of a simple automated optimization routine [[5](#_ENREF_5), [6](#_ENREF_6)]. Specifically, the agreement between the model and data is quantified by calculating the root mean square error (RMSE) for each of the datasets in panels B-O in Fig. S1. Fig. S2A presents a direct comparison of model and data. The overall error is the average across the datasets. Since the individual datasets have various units and ranges, the RMSE of each dataset is normalized to the RMSE of a simulation with the initial/starting parameter values prior to averaging to compute the overall RMSE. Other methods of quantifying the model performance (e.g. standardized rms error statistics, [[7](#_ENREF_7)]) were evaluated but found not to produce different overall results. The optimization routine minimizes the error by varying the parameters identified in Table S1. The routine starts by running the model using initial/starting values, which reflect an initial manual calibration. Then it proceeds down the list of parameters in random order and randomly increases or decreases its value (within the literature range). It then runs the model three times with different seed values for the random number generator and calculates the average overall RMSE. The symbols in Fig. S2B show the overall RMSE for each parameter perturbation, which also provides some insight into the effect of individual parameter perturbations. If the model performance increases, it retains the new parameter value and if not, it reverts to the previous value. Then it proceeds with the next parameter. This leads to a decrease in model error with the number of simulations. The line in Fig. S2B shows the best overall RMSE during the course of the optimization. The optimization routine is run twice, with different seed values of the random number generator. Those simulations are reflected by the different colors in Fig. S2B. Both simulations converge to the same overall RMSE and they have very close parameter values.

S2. Additional discussion of model results

*Growth and damage*

The model is compared to observations from the literature in Fig. S1 [[8-16](#_ENREF_8)]. Microcolonies were grown using a high-throughput microscopy assay and growth rates were estimated based on the change in colony area, which correlates with cell count. The observed growth rate of microcolonies varies, as illustrated by three representative colonies (Fig. S1A) and the growth rate distribution for all colonies (Fig. S1B). The model distribution is very close and not easily distinguishable from the observed one at the scale of the figure (Fig. S1B). The damage vs. age plot is discussed in the main text (Fig. S1C, Fig. 2A). The model reproduces these patterns. Fig. S1A shows that the growth rate is heritable over several generations. When the model is allowed to continue (without nutrient limitation), the growth rates of the colonies will eventually become the same and approach the mean of all colonies (Fig. S1B, Fig. S3). Fig. S1B shows that the growth rate is heterogeneous. The distribution is for colonies with up to about 100 cells. The growth rates of individual cells within a colony varies as well (Fig. S4).

*Tsl1 and trehalose*

There is significant uncertainty associated with average protein levels of Tsl1 and Tps3, which likely reflect different experimental protocols, but the model is within the range of observations (Fig. S1D). The expression of Tsl1 is discussed in the main text (Figs. S1E&F, Figs. 2B&C). Sorting out the top 1% of Tsl1-expressing cells and counting bud scars manually also shows that Tsl1 expression increases with age (Fig. S1G). The model under-predicts the population fraction of older cells, which may be due to limitations of the adopted replication model (Fig. S5). However, the model reproduces the observation that higher Tsl1 cells are older, which is what is important here. Colonies exhibit a negative correlation between Tsl1 expression and growth rate (Fig. S1H). Performing the microcolony growth rate assay on sub-populations sorted by Tsl1 expression also shows this (Fig. S1I). Sorting cells, letting them grow for 40 generations and then performing the growth rate assay shows that this difference disappears and therefore is not genetic (Fig. S1J). The model reproduces this observed pattern, which is because Tsl1 expression increases and the growth rate decreases with damage (Eqs. 2&4). The causal link between Tsl1 and growth rate (*e_Tsl1_* > *f_s_* > growth) is there, but it does not contribute significantly to the correlation between Tsl1 and growth rate (Fig. S6). The amount of trehalose synthesized in the model is generally consistent with unstressed, fast-growing cells (Fig. S1K). A Tsl1 knockout strain produces less trehalose than the wildtype (Fig. S1L).

*Heat shock*

The survival vs. Tsl1 expression is discussed in the main text (Fig. S1M, Fig. 2D). The same pattern is seen when colonies were heat shocked and survival, defined as at least one cell of the colony surviving, was observed. Survival probability of microcolonies correlates positively with Tsl1 expression (Fig. S1N) and negatively with growth rate (Fig. S1O). The Tsl1 knockout strain has lower survival than the wild-type (Fig. S1O). The model reproduces these patterns. Damage correlates positively with Tsl1/Tps3 expression (Eq. 4), trehalose (Eq. 3) and survival (Eq. 5), and negatively with growth rate (Eq. 2). Note that colonies with higher growth rates have more cells at the time the heat shock is administered and thus have a higher chance of survival. This counteracts the pattern seen in Fig. S1O. Thus, the relation between growth rate and survival of individual cells has to be stronger than that shown in Fig. S1O ([[8](#_ENREF_8)], see Fig. S7). This distinction is important, because in our competition experiments individual cells, not colonies, compete. The individual-based model naturally links survival at the individual and colony levels.

Fig. S1. Model-data comparison. (A) Growth of three representative colonies. (B) Growth rate distribution of colonies. (C) Damage (*m_D_* / *m*) vs. age (*n_B_*). Error bars on data are ± 1 standard deviation. (D) Protein expression levels (*e_Tsl1_*, *e_Tps3_*). (E) Tsl1 expression (*e_Tsl1_*) distribution of cells. (F) Age (*n_B_*) vs. Tsl1 expression (*e_Tsl1_*). Error bars on data are ± 1 standard error of the mean (SEM). (G) Age (*n_B_*) distribution of cells for all and top 1% of Tsl1-expressing cells. (H) Tsl1 expression (*e_Tsl1_*) vs. growth rate. Error bars on data are ± 1 SEM. (I) Growth rate distribution of colonies of various Tsl1-sorted fractions. (J) Growth rate distribution of colonies of same fractions after 42 generations of growth. (K) Trehalose fraction (*m_T_* / *m*). (L) Trehalose levels (*m_T_* / *m*) in wildtype (WT) vs. Tsl1 knockout (KO) strains. (M) Heat shock survival of various Tsl1-sorted fractions. Error bars on data are ± 1 SEM. (N) Heat shock survival vs. Tsl1 expression (*e_Tsl1_*). (O) Heat shock survival of wildtype (WT) and Tsl1 knockout (KO) strains vs. growth rate. "d" and "m" indicate data and model, respectively. "a.u." is arbitrary units. Data sources and notes: Panels A, B, E, F, G, H, I, J, M, N, O: [[8](#_ENREF_8)]; C: [[9](#_ENREF_9)], protein carbonyl levels and bud scars; D: d1: [[10](#_ENREF_10)], d2/3: [[11](#_ENREF_11)] YEPD/SD; K: d1: [[12](#_ENREF_12)], d2: [[13](#_ENREF_13)], d3: [[14](#_ENREF_14)], d4: [[15](#_ENREF_15)]; L: d1 [[16](#_ENREF_16)], d2: [[13](#_ENREF_13)] (see Table S1, footnote C for conversions and notes). For details of experiments used to generate the data the reader is referred to the source publications.

Fig. S2. Automatic calibration using optimization routine [[5](#_ENREF_5), [6](#_ENREF_6)]. (A) Quantitative model vs. data comparison. Series names refer to panels in Fig. S1. Error is calculated as the average (across panels B-O in Fig. S1) normalized root mean square error (RMSE). The optimization routine minimizes the error by varying the parameters identified in Table S1. (B) Error as a number of simulations. Points are individual simulations and lines are the lowest error achieved so far. Different colors correspond to optimizations with different seed values of the random number generator.

Fig. S3. Additional model results for three representative colonies (same as in Fig. S1A). (A) Growth of colonies over longer time period. Colony growth rates, estimated as slope over 15-20 hours, are 0.41 h^-1^ for all three colonies. Mean of all colonies (Fig. S1B) is 0.41 h^-1^ (*µ_n_*, see caption Fig. S5). (B) Selected model variables of colonies illustrating mechanism of heterogeneity and inter-generation memory. (C) Selected model variables of individual cells in three microcolonies.

Fig. S4. Growth rate distributions of microcolonies and cells. (A) Comparison of different methods for calculating colony growth rate. Area: based on change of colony area (*µ_a_*), same as in Fig. S1B. Cells: based on change of number of cells in colony (*µ_n_*). Rate: average biomass growth rate (*µ*, Eq. 1). (B) Reproducibility of results. Growth rate (*µ_a_*) distribution of three runs with different seed values for the random number generator (*RS*). (C) Evolution of growth rate distribution during experiment. Average and instantaneous *µ*. (D) Colony vs. cells. Instantaneous *µ*. (E) Growth rate distribution of cells within a microcolony. Selected microcolonies (same as in Figs. S1A, S3). Instantaneous *µ* at 9h.

Fig. S5. Age distribution. Comparison of observations by Levy et al. [[8](#_ENREF_8)] (same as Fig. S1G, all), this model and Vanoni et al. [[17](#_ENREF_17)] model (Table 3, *T* = 104 min). Note that both models underestimate the fraction of older cells.

Fig. S6. Mechanisms underlying the correlation between Tsl1 expression and growth rate. (A) Tsl1 expression vs. growth rate. Same as Fig. S1H. (B) Growth rate limitation terms. Dam: Damage term in Eq. 2. TS: Trehalose synthesis term (1 - *f_s_*) in Eq. 1a. (C) Trehalose synthesis enzyme levels. (D) Damage (*m_D_* / *m*). (E) Age (*n_B_*).

Fig. S7. Heat shock survival of (left column) microcolonies and (right column) cells, binned by growth rate. Panel A1 is same as Fig. S1O. Figure illustrates how age translates to survival, and how the pattern is stronger for individual cells. Growth rates of microcolonies estimated from change in colony area (*µ_a_*). Growth rate of cells is biomass-based (*µ*, Eq. 2) growth rate.

Fig. S8. Source of heat shock tolerance heterogeneity. (A) Simulations to investigate contribution of various mechanisms of heterogeneity [[6](#_ENREF_6), [18](#_ENREF_18)]. “base” is the base case. “dam” uses equal damage split fraction (*s_d_* = 0.5). “scar” has no effect of bud scar (on *m_b_* or *k_d_*). “mass” uses equal mass split fraction (*f_m,r_* = 2). “mb,0”, “fr,Tsl1”, “fr,Tps3”, “um,g” and “nd” have assigned CV = 0 for *m_b,0_*, *f_r,Tsl1_*, *f_r,Tps3_*, *µ_m,g_* and *n_d_*, respectively. (A) Distribution of normalized heat shock tolerance (*H_t_*). Same as Fig. 2E. (B) Heterogeneity quantified as variance. (C) Heterogeneity network. Same as Fig. 2F, but line weights not based on contribution. All variables defined in main text. Processes: h = heat shock tolerance (Eq. 5), m = total mass summation (*m* = *m_X_* + *m_D_* + *m_T_*), T = trehalose mass balance (Eq. 1c), D = damage mass balance (Eq. 1b), X = structural mass balance (Eq. 1a), f = fraction trehalose synthesized (Eq. 3), e = enzyme summation (*e_s_* = *e_Tsl1_* + *e_Tps3_*), 3 = Tps3 expression (like Eq. 4), 1 = Tsl1 expression (Eq. 4), d = damage rate ($k_{d}=a_{d} n_{B}^{b_{d}}$), b =budding size (see text), R = replication (if *m_X_* > *m_r_* then…), r = replication size (*m_r_* = *f_m,r_* *m_b_*). Sources of heterogeneity: DAM = unequal division of damage, SCAR = bud scaring, MASS = unequal division of mass, and *R-m_b,0_*, *R-f_r,Tsl1_*, *R-f_r,Tps3_*, *R-µ_m,g_* and *R-n_d_* = stochastic variability in *m_b,0_*, *f_r,Tsl1_*, *f_r,Tps3_*, *µ_m,g_* and *n_d_*.

Fig. S9. Competition experiment. Strain 1 is calibration (the same strain shown in Fig. 2 and S1, mBc, see Table S2). Strain 2 has constant expression and no heterogeneity (mBx, see Table S2). *H_a_* = 0.6, *F_h_* = 0.14 d^-1^. The figure illustrates how a bet hedging strategy is more beneficial.

Fig. S10. Additional competition simulations. Tsl1/Tps3 expression parameters of winning strain (as in Fig. 3B). (A) Excluding age-dependent stress resistance (*e_a,Tsl1_* = 0). (B) Excluding age-dependent stress resistance and stochasticity (*e_a,Tsl1_* = 0, *f_r,Tsl1,CV_* = 0).

Fig. S11. Diagnostic simulation to understand mechanism underlying fitness advantage of age-correlated stress resistance. The two strains have no trehalose production. For Strain 1, older cells (*n_B_* = 3) are handicapped. For Strain 2, younger cells (*n_B_* = 0) are handicapped. Handicap involves periodic elimination of a number of cells (50% of the number of cells in the group with lower number, every 14 days). The figure illustrates that eliminating or slowing older cells is less harmful than younger cells.

**Table S1. Model parameters**

| **Symbol** | **Units** | **Mean [CV] (a)** | **Notes** |
| --- | --- | --- | --- |
| *µ_m,g_* | h^-1^ | 0.55* [0.10*] | = 0.44-0.45 (b) |
| *K_g_* | g L^-1^ | 0.10 | = 0.1 (b) |
| *K_d_* | - | mAc: 0.19*, mBc: 0.14* |  |
| *n_d_* | - | 1.6* [1.0*] |  |
| *m_b,0_* | pg dry cell^-1^ | 8.0 [0.25] | (c) [= 0.20 (d), 0.25, 0.34 (e)] |
| *a_m_* | - | 0.20 | = 0.20 (0.15-0.20) (d) |
| *b_m_* | - | 0.50 | = 0.50 (d) |
| *f_m,r_* | - | 1.7 | = 1.7 (1.5-1.8) (d) |
| *a_d_* | d^-1^ | 1.0* |  |
| *b_d_* | - | 0.32* | > 0 (f), = 0.32-0.53 (g) |
| *s_d_* | - | 0.83 | = 0.84, 0.90(h), 0.79±0.07 (i) |
| *e_c,Tsl1_* | µM | 0.093* |  |
| *e_a,Tsl1_* | µM | 0.67* |  |
| *K_Tsl1_* | - | 6.0* |  |
| *f_r,Tsl1_* | - | 1.0 [3.0*] |  |
| *e_c,Tps3_* | µM | 0.0* × *e_c,Tsl1_* |  |
| *e_a,Tps3_* | µM | 160* × *e_a,Tsl1_* |  |
| *K_Tps3_* | - | = *K_Tsl1_* |  |
| *f_r,Tps3_* | - | 1.0 [0.10*×Tsl1] | [Tps3/Tsl1 = 0.015-0.10 (j)] |
| *f_m,s_* | % | 16 | population average: 2.0 (k), 20 (l) |
| *K_s_* | µM | 2.5* |  |
| *n_s_* | - | 2.0* |  |
| *H_a_* | - | varies | varies by experiment |
| *F_h_* | d^-1^ | varies | varies by experiment |
| *K_h_* | - | 0.040* | = 0.038-0.19 (m) |
| *V_R_* | L | varies | varies by experiment |
| *Q* | L h^-1^ | varies | varies by experiment |
| *G_IN_* | g L^-1^ | 20 | = 20 (n) |
| *Y_g_* | - | 0.15 | = 0.15-0.16 (b) |
|  |  |  |  |

(a) Parameters marked with * were calibrated with the help of an automated optimization routine [[5](#_ENREF_5), [6](#_ENREF_6)]. Value in [] is randomization coefficient of variation (CV). mAc, mBc, etc. refer to different model strains, see Table S2.

(b) *µ_1_*, *K_1_*, *Y_1_*, [[19](#_ENREF_19)]. The formulation in the reference does not include damage limitation, so *µ_m,g_* > *µ_1_*.

(c) *m_b,0_* was calibrated manually to get *m_AVE_* at high *µ* (A).

(d) [[17](#_ENREF_17)]; *a_m_* is *Q*; *b_m_* is *a* in reference; *f_m,r_* based on *T* and *T_B_* in Table 1 of reference.

(e) [[20](#_ENREF_20)]; daughter, parent cells, Table 1 of reference.

(f) Based on higher ROS observed in older cells [[21](#_ENREF_21)].

(g) Based on superoxide and hydrogen peroxide levels in mother (10-12 generations old) and daughter cells, WT, separated [[22](#_ENREF_22)].

(h) Based on 3.6, 6-fold lower damage density in daughters [[9](#_ENREF_9)] (B).

(i) Based on 0.39±0.17 ratio bud/mother [[23](#_ENREF_23)] (B).

(j) Based on single-cell protein abundance observations, "DM" measure, synthetic dextrose media, [[11](#_ENREF_11)].

(k) In late stationary phase, 1.8 µg (10^8^ cells)^-1^ [[13](#_ENREF_13)]; converted using *m_AVE_* at low *µ* (A).

(l) Max. observed [[14](#_ENREF_14)]; converted using *m_AVE_* at low *µ* (A).

(m) Based on enzyme activity vs. trehalose [[24](#_ENREF_24)], Fig. 1, converted from 100-500 mM using 0.60 cell water mass fraction [[25](#_ENREF_25)] and 0.25 vacuole volume fraction [[24](#_ENREF_24)].

(n) Synthetic Complete Media.

(A) *m_AVE_* = 10.6 pg dry cell^-1^ at high *µ* = 0.20 h^-1^, 17.4 pg dry cell^-1^ at low *µ* = 0.033 h^-1^ [[12](#_ENREF_12)].

(B) Converted using mother mass split fraction 1 / *f_m,r_* (see table entry).

(C) Conversions and notes. Fig. S1K, d1: [[12](#_ENREF_12)], at *µ* = 0.20 h^‑1^, 0 fmol glucose cell^-1^, d2: [[13](#_ENREF_13)], late exponential, 0.14 µg (10^8^ cells)^-1^, converted using *m_AVE_* at high *µ* (A), d3: [[14](#_ENREF_14)], at consumption rate > 31 fmol cell^-1^ h^-1^, 0.4-1.3 fmol glucose cell^-1^, converted using *m_AVE_* at high *µ* (A), d4: [[15](#_ENREF_15)], dilution rate = 0.1 h^-1^. Fig. S1L: d1: stationary phase, [[16](#_ENREF_16)]. d2: all growth phases, [[13](#_ENREF_13)].

**Table S2. Strain summary – Tsl1/Tps3 expression parameters (a)**

| **Strain(s)** | ***e_c,Tsl1_*** | ***e_a,Tsl1_*** | ***f_r,Tsl1,CV_*** | **Description** |
| --- | --- | --- | --- | --- |
| mAc | Table S1 | Table S1 | Table S1 | Calibration A |
| mBc | Table S1 | Table S1 | Table S1 | Calibration B |
| mBk | 0 | 0 | 0 | Calibration B without Tsl1 |
| mB*i* | 0-4.0 | 0-8.0 | 0-50 | Competition range |
| mBx | 5.0 | 0 | 0 | No bet hedging |
|  |  |  |  |  |

(a) Units in Table S1.

**Table S3. Strain summary – model vs. data**

| **Figure(s)** | **Data strain(s)** | **Model strain(s) (a)** |
| --- | --- | --- |
| S1A | YFR054C [[8](#_ENREF_8)] | mAc |
| S1B | YFR054C, YHR095W [[8](#_ENREF_8)] | mAc |
| 2B, 2C, 2D, 2E, 2F, S1E, S1F,  S1G, S1H, S1I, S1J, S1M, S1N | Tsl1-GFP [[8](#_ENREF_8)] | mBc |
| S1O | Tsl1-GFP, Tsl1Δ-mCherry [[8](#_ENREF_8)] | mBc, mBk |
| 2A, S1C | BY4741 [[9](#_ENREF_9)] | mAc |
| S1K | d1: CEN-PK113-7D [[12](#_ENREF_12)],  d2: BY4742 [[13](#_ENREF_13)],  d3: SU32 [[14](#_ENREF_14)],  d4: LBG H 1011 [[15](#_ENREF_15)] | mAc |
| S1L | d1: YSH6.127.-1C, YSH6.127.-4B [[16](#_ENREF_16)]  d2: BY4742, BY4742 tsl1Δ [[13](#_ENREF_13)] | mBc, mBk |
| 3, S11 | - | mB*i* (b) |
| S3, S4 | - | mAc |
| S6, S7 | Tsl1-GFP [[8](#_ENREF_8)] | mBc |
| S8 | Tsl1-GFP, Tsl1Δ-mCherry [[8](#_ENREF_8)] | mBc, mBk |
| S9 | - | mBc, mBx |
| S10 | - | mBc |
| S12 | - | mBc |
|  |  |  |

(a) See Table S2.

(b) mB*i* (*i* = 1 ... *n*) are strains with different Tsl1/Tps3 expression parameters.

**References**

1. Hellweger FL, Kravchuk ES, Novotny V, Gladyshev MI: **Agent-Based Modeling of the Complex Life Cycle of a Cyanobacterium (Anabaena) in a Shallow Reservoir**. *Limnology and Oceanography* 2008, **53**(4):1227-1241.

2. Hellweger FL, Bucci V: **A bunch of tiny individuals—Individual-based modeling for microbes**. *Ecological Modelling* 2009, **220**(1):8-22.

3. Hellweger FL: **Spatially explicit individual-based modeling using a fixed super-individual density**. *Computers & Geosciences* 2008, **34**(2):144-152.

4. Hellweger FL: **Carrying photosynthesis genes increases ecological fitness of cyanophage *in silico***. *Environmental Microbiology* 2009, **11**(6):1386-1394.

5. Bucci V, Majed N, Hellweger FL, Gu AZ: **Heterogeneity of Intracellular Polymer Storage States in Enhanced Biological Phosphorus Removal (EBPR) – Observation and Modeling**. *Environmental Science & Technology* 2012, **46**(6):3244-3252.

6. Fredrick ND, Berges JA, Twining BS, Nuñez-Milland D, Hellweger FL: **Exploring Mechanisms of Intracellular P Heterogeneity in Cultured Phytoplankton using Agent Based Modeling**. *Applied and Environmental Microbiology* 2013.

7. Perretti CT, Munch SB, Sugihara G: **Model-free forecasting outperforms the correct mechanistic model for simulated and experimental data**. *Proceedings of the National Academy of Sciences* 2013.

8. Levy SF, Ziv N, Siegal ML: **Bet Hedging in Yeast by Heterogeneous, Age-Correlated Expression of a Stress Protectant**. *PLoS Biol* 2012, **10**(5):e1001325.

9. Aguilaniu H, Gustafsson L, Rigoulet M, Nyström T: **Asymmetric Inheritance of Oxidatively Damaged Proteins During Cytokinesis**. *Science* 2003, **299**(5613):1751-1753.

10. Ghaemmaghami S, Huh W-K, Bower K, Howson RW, Belle A, Dephoure N, O'Shea EK, Weissman JS: **Global analysis of protein expression in yeast**. *Nature* 2003, **425**(6959):737-741.

11. Newman JRS, Ghaemmaghami S, Ihmels J, Breslow DK, Noble M, DeRisi JL, Weissman JS: **Single-cell proteomic analysis of S. cerevisiae reveals the architecture of biological noise**. *Nature* 2006, **441**(7095):840-846.

12. Silljé HHW, Paalman JWG, ter Schure EG, Olsthoorn SQB, Verkleij AJ, Boonstra J, Verrips CT: **Function of Trehalose and Glycogen in Cell Cycle Progression and Cell Viability in Saccharomyces cerevisiae**. *Journal of Bacteriology* 1999, **181**(2):396-400.

13. Bandara A, Fraser S, Chambers PJ, Stanley GA: **Trehalose promotes the survival of Saccharomyces cerevisiae during lethal ethanol stress, but does not influence growth under sublethal ethanol stress**. *FEMS Yeast Research* 2009, **9**(8):1208-1216.

14. Silljé HH, ter Schure EG, Rommens AJ, Huls PG, Woldringh CL, Verkleij AJ, Boonstra J, Verrips CT: **Effects of different carbon fluxes on G1 phase duration, cyclin expression, and reserve carbohydrate metabolism in Saccharomyces cerevisiae**. *Journal of Bacteriology* 1997, **179**(21):6560-6565.

15. Küenzi MT, Fiechter A: **Changes in carbohydrate composition and trehalase-activity during the budding cycle of *Saccharomyces cerevisiae***. *Archives of Microbiology* 1969, **64**(4):396-407.

16. Bell W, Sun W, Hohmann S, Wera S, Reinders A, De Virgilio C, Wiemken A, Thevelein JM: **Composition and Functional Analysis of the Saccharomyces cerevisiae Trehalose Synthase Complex**. *Journal of Biological Chemistry* 1998, **273**(50):33311-33319.

17. Vanoni M, Vai M, Popolo L, Alberghina L: **Structural heterogeneity in populations of the budding yeast Saccharomyces cerevisiae**. *Journal of Bacteriology* 1983, **156**(3):1282-1291.

18. Bucci V, Nunez-Milland D, Twining B, Hellweger F: **Microscale patchiness leads to large and important intraspecific internal nutrient heterogeneity in phytoplankton**. *Aquat Ecol* 2012, **46**(1):101-118.

19. Jones KD, Kompala DS: **Cybernetic model of the growth dynamics of Saccharomyces cerevisiae in batch and continuous cultures**. *Journal of Biotechnology* 1999, **71**(1–3):105-131.

20. Wheals AE: **Size control models of Saccharomyces cerevisiae cell proliferation**. *Molecular and Cellular Biology* 1982, **2**(4):361-368.

21. Laun P, Pichova A, Madeo F, Fuchs J, Ellinger A, Kohlwein S, Dawes I, Fröhlich K-U, Breitenbach M: **Aged mother cells of Saccharomyces cerevisiae show markers of oxidative stress and apoptosis**. *Molecular Microbiology* 2001, **39**(5):1166-1173.

22. Erjavec N, Nyström T: **Sir2p-dependent protein segregation gives rise to a superior reactive oxygen species management in the progeny of Saccharomyces cerevisiae**. *Proceedings of the National Academy of Sciences* 2007, **104**(26):10877-10881.

23. Erjavec N, Larsson L, Grantham J, Nyström T: **Accelerated aging and failure to segregate damaged proteins in Sir2 mutants can be suppressed by overproducing the protein aggregation-remodeling factor Hsp104p**. *Genes & Development* 2007, **21**(19):2410-2421.

24. Hottiger T, De Virgilio C, Hall MN, Boller T, Wiemken A: **The role of trehalose synthesis for the acquisition of thermotolerance in yeast**. *European Journal of Biochemistry* 1994, **219**(1-2):187-193.

25. Illmer P, Erlebach C, Schinner F: **A practicable and accurate method to differentiate between intra- and extracellular water of microbial cells**. *FEMS Microbiology Letters* 1999, **178**(1):135-139.
